# Welcome to the readers

**DOI:** 10.4103/0972-2327.78040

**Published:** 2011

**Authors:** Sanjeev V. Thomas

**Affiliations:** Editor, Annals of Indian Academy of Neurology, Room 1409, Department of Neurology, Sree Chitra Tirunal Institute for Medical Sciences and Technology, Trivandrum - 695 011, India. E-mail: editor@annalsofian.org

Let me extend a warm welcome to all the readers as we come out with the first issue of the journal for the year 2011. AIAN has come a long way in the past 13 years, with steady improvement in its standards and readership. This issue of the journal carries several important articles. The review article on management of provoked seizures discusses several practically relevant issues. There is a renewed global interest to prevent the occurrence of epilepsy. One-third of epilepsy cases in certain geographic areas of the world are due to acquired preventable causes such as head injury, neurocysticercosis (NCC), other types of central nervous system (CNS) infections and obstetrical causes. Lifestyle modifications, hygiene, trauma protection, identification of high-risk cases and prompt management to minimize cerebral damage have an important role to eventually prevent epilepsy. Physicians, paediatricians and neurologists are often called upon to manage acute symptomatic seizures and provoked seizures. The article on management of provoked seizures provides an evidence base for the dos and donts in the management of provoked seizures. I hope the readers will find this article very helpful. Attention-deficit hyperactive disorder (ADHD) is an important co-morbidity in children with epilepsy. The prevalence of ADHD in children with epilepsy is several times higher than that in the general population. Parents of children with ADHD occasionally confuse between some of the behavioral features of ADHD with postictal or ictal behavior. Adverse effects of medications can aggravate both disorders. Some of the medications recommended for ADHD may adversely influence seizure control. Similarly, some of the antiepileptic drugs can also aggravate ADHD. Antiepileptic drugs like carbamazepine can be beneficial to both conditions. It is important that physicians treating children with epilepsy are vigilant about ADHD in their patients. They should be familiar with standard methods to ascertain the presence of ADHD and monitor the progress of the condition under treatment. NCC is the most important acquired cause of epilepsy in several parts of India. It is unfortunate that ignorance about how the disease spreads, poor hygiene, improper sewerage disposal, pig rearing and several socio-cultural factors influence the occurrence of NCC and the resultant epilepsy. The survey on the knowledge of epilepsy published in this issue would be one of the foundation papers in the prevention of epilepsy due to NCC. Meniere’s disease is a very common condition encountered by neurologists, yet there is much less discussion on this topic in the journals. The triad of tinnitus, hearing impairment and vertigo constitutes Meniere’s disease. There had been considerable progress in the evaluation of vertigo, particularly in the electrophysiological and imaging domains. The article on Meniere’s disease in this journal discusses some of these new developments. Idiopathic intracranial hypertension (IIH) had been labelled as benign for several decades. Nevertheless, some of these patients carry a higher risk of significant visual impairment or total visual loss when followed-up for longer periods of time. The paper on long-term visual outcome of IIH attempts to document the long-term outcome of such patients in India. About 10% of the patients with IIH experienced significant visual impairment or worsening when followed-up for 5 years. It is important that patients with IIH are kept under follow-up even after an initial improvement and warned about the possible late visual deterioration. Spasticity is one of the major factors limiting recovery or rehabilitation in patients with stroke. Several degenerative disorders of the CNS are characterized by significant spasticity. Systematic assessment and quantification of spasticity is very important while recommending appropriate rehabilitation plans. One of the papers in this issue discusses the methods to evaluate spasticity in the upper and lower limbs. The technical notes on single-fiber EMG (SFEMG) in this issue would be particularly helpful for those who intend to start this service in their laboratories. SFEMG is an art and science that has immense application in the field of neuromuscular disorders. There are several other important papers, case reports and images in this issue that the readers would find useful.

